# The Gut Microbiome in Pouchitis: A Narrative Review of Pathogenesis and Therapeutic Modulation

**DOI:** 10.3390/nu18172844

**Published:** 2026-08-31

**Authors:** Sara Deleu, Sien Hoekx, João Sabino

**Affiliations:** 1 Department of Chronic Diseases, and Metabolism (CHROMETA), KU Leuven, 3000 Leuven, Belgium; joao.sabino@uzleuven.be; 2 Department of Gastroenterology and Hepatology, University Hospitals Leuven, KU Leuven, 3000 Leuven, Belgium

**Keywords:** inflammatory bowel diseases (IBD), pouchitis, gut microbiome, pathogenesis, therapeutic modulation

## Abstract

Pouchitis is the most common long-term complication following restorative proctocolectomy with ileal pouch-anal anastomosis (IPAA) for ulcerative colitis (UC), with a cumulative prevalence of up to 50% within the first two years after IPAA surgery. Accumulating evidence implicates gut microbial dysbiosis in the pathogenesis of pouch inflammation, supported by characteristic microbial alterations and the clinical responsiveness of pouchitis to microbiota-directed therapies such as antibiotics and probiotics. This narrative review synthesizes evidence mainly published in the last decade on microbiota alterations in pouchitis, the mechanisms of microbiota–host interactions that may contribute to inflammation, and the clinical evidence linking the gut microbiota to disease development and progression. Therapeutic strategies for modulating the pouch microbiota, including probiotics, prebiotics, dietary interventions, antibiotics, and fecal microbiota transplantation, are critically evaluated. Finally, future directions in multi-omics profiling, precision microbiome medicine, and next-generation live biotherapeutics are discussed. Overall, current evidence supports antibiotics as the first-line treatment for pouchitis, although it does not restore microbial eubiosis. Among microbiota-targeted interventions, the eight-strain De Simone formulation has the strongest evidence for prevention, whereas faecal microbiota transplantation remains investigational and is not currently supported outside clinical trials. Further research integrating multi-omics and microbiome profiling is needed to better define disease mechanisms and enable personalized microbiome-based approaches to prevention and treatment.

## 1. Introduction

Restorative proctocolectomy with ileal pouch-anal anastomosis (IPAA) remains the surgical procedure of choice for patients with ulcerative colitis (UC) who require colectomy due to medically refractory disease or the development of colorectal neoplasia [1,2]. A meta-analysis of 26 population-based cohorts reported a contemporary 1-, 5-, and 10-year risks of colectomy of 2.8%, 7.0%, and 9.6%, respectively [3]. While IPAA preserves intestinal continuity and is associated with high long-term quality of life, the ileal pouch is susceptible to a range of structural, functional, and inflammatory complications that necessitate ongoing surveillance [1].

Pouchitis, nonspecific inflammation of the ileal pouch mucosa, is the most common long-term complication of IPAA [2,4]. The cumulative incidence of pouchitis is substantial: up to 50% of patients develop pouchitis within the first 2 years after IPAA, and up to 80% experience at least one episode over their lifetime [5,6]. This was confirmed by a meta-analysis of 59 studies (*n* = 18,117) which reported an overall prevalence of 31%, with acute pouchitis occurring in 18% and chronic pouchitis in 13% [7]. In a large US population-based cohort, the incidence of pouchitis increased from 58% at 1 year to 72% at 10 years after IPAA, with 14% of patients ultimately requiring advanced immunosuppressive therapies [8]. Differences among these estimates likely reflect variation in study design, case definitions, ascertainment methods, and duration of follow-up, which complicate direct comparisons across studies. Notably, the incidence of pouchitis within the first 2 years after IPAA may have increased in recent decades, even as colectomy rates have declined [3,6].

Clinically, pouchitis presents with increased stool frequency, fecal urgency, abdominal cramping, and occasionally bloody stools [2,4]. Diagnosis requires endoscopic confirmation of inflammation, and disease activity is most commonly assessed using the pouchitis disease activity index (PDAI) or its modified version (mPDAI), although neither instrument has been formally validated [2,9]. Functional outcomes after IPAA can be separately assessed using the Öresland Score, a 0–15-point scale evaluating stool frequency, urgency, and continence [10]. Pouchitis represents a disease spectrum, classified by duration (acute vs. chronic), disease pattern (infrequent, relapsing, or continuous), and response to antibiotics (responsive, dependent, or refractory) [11,12]. This classification framework, endorsed by the International Ileal Pouch Consortium, has been applied in clinical trial design and guides therapeutic decision-making [11].

The pathogenesis of pouchitis is multifactorial, involving complex interactions between genetic susceptibility, the gut microbiota, dysregulated host immunity, fecal stasis, surgical technique, and ischaemia [12]. However, the gut microbiota has emerged as a central factor. The ileal pouch undergoes colonic metaplasia after IPAA, acquiring a colon-like microbial community that, in genetically susceptible individuals, becomes dysbiotic and triggers an aberrant immune response [11,12,13]. Primary or idiopathic pouchitis is believed to result from this abnormal immune response to luminal dysbiosis, without identifiable pathogenic organisms [12]. The strongest clinical evidence for a microbial role comes from the responsiveness of acute pouchitis to antibiotic therapy and the efficacy of select probiotics in preventing pouchitis and maintaining remission [6,14]. Pouchitis occurs almost exclusively in UC patients and only rarely in those with familial adenomatous polyposis (FAP) who undergo the same surgical procedure, further implicating disease-specific host–microbiota interactions [15].

Risk factors for pouchitis include primary sclerosing cholangitis (PSC), pre-colectomy exposure to anti-TNF therapy or vedolizumab, smoking or nicotine dependence, and indeterminate colitis [5,8,16]. Approximately 17% of patients develop chronic pouchitis with a relapsing-remitting course, and 10% develop Crohn’s-like disease of the pouch [17]. Chronic antibiotic-refractory pouchitis remains a significant therapeutic challenge, often requiring escalation to biologic agents such as vedolizumab, anti-TNF inhibitors, ustekinumab, or JAK inhibitors [6]. In the landmark EARNEST trial (*n* = 102), vedolizumab demonstrated superiority over placebo for chronic pouchitis, with clinical remission rates of 31% vs. 10% at week 14 and 35% vs. 18% at week 34 [2].

Despite these advances, the evidence base for pouchitis management remains limited, derived primarily from retrospective observational studies and small cohorts, resulting in substantial practice variability [6]. The recognition that the microbiota is both a pathogenic driver and a therapeutic target has catalyzed a growing body of research into microbiota-directed interventions, from traditional probiotics and antibiotics to fecal microbiota transplantation and next-generation live biotherapeutics. While several recent reviews have explored the role of the microbiota in pouch-related disorders, important gaps remain. Most have focused primarily on bacterial dysbiosis and established therapeutic approaches, with limited attention to cross-kingdom interactions involving the mycobiome and virome, nutritional influences, emerging microbiome-directed therapies, and non-invasive biomarkers. This review is guided by the concept that the gut microbiota may act as a contributor to disease pathogenesis, a modulator of disease activity, and a therapeutic target in pouchitis. Specifically, this review aims to address four key questions: (1) What microbiota alterations characterize pouchitis? (2) Through which mechanisms does the microbiota contribute to pouch inflammation? (3) What evidence supports a pathogenic role of the microbiota in disease development and progression? (4) What is the current and future role of microbiota-targeted therapies? Therefore, this review provides a comprehensive synthesis of the current evidence on the role of the gut microbiota in pouchitis pathogenesis and its therapeutic modulation, offering a broader and more clinically relevant framework for understanding and managing pouch disorders.

## 2. Methods

This narrative review was conducted through a comprehensive search of the published literature to synthesize current evidence on the role of the gut microbiota in the pathogenesis and therapeutic modulation of pouchitis. PubMed was searched from inception through 30 June 2026 for the following terms and their combinations: “pouchitis,” “ileal pouch,” “ileal pouch-anal anastomosis,” “IPAA,” “restorative proctocolectomy,” “microbiota,” “microbiome,” “dysbiosis,” “gut bacteria,” “fecal microbiota transplantation,” “FMT,” “probiotics,” “prebiotics,” “synbiotics,” “antibiotics,” “diet,” “dietary intervention,” “short-chain fatty acids,” “butyrate,” “bile acids,” “metabolomics,” “metagenomics,” “multi-omics,” “live biotherapeutics,” “engineered bacteria,” and “bacteriophage.” Boolean operators (AND, OR) were used to combine search terms across thematic domains.

Studies were eligible for inclusion if they reported on (a) microbial composition or function in patients with ileal pouches, with or without pouchitis; (b) microbiota–host interactions relevant to pouch inflammation, including barrier function, immune activation, and microbial metabolite pathways; (c) clinical outcomes of microbiota-directed therapies in pouchitis, including antibiotics, probiotics, prebiotics, dietary interventions, and fecal microbiota transplantation; or (d) emerging therapeutic approaches targeting the pouch microbiota, including live biotherapeutics and engineered microbial consortia.

Priority was given to clinical practice guidelines from major gastroenterological societies (American Gastroenterological Association, American Society of Colon and Rectal Surgeons, European Crohn’s and Colitis Organisation, International Ileal Pouch Consortium), systematic reviews and meta-analyses, randomized controlled trials, and prospective cohort studies. Retrospective observational studies, case series, and preclinical studies were included when higher-level evidence was unavailable or when they provided unique mechanistic insights. Conference abstracts were included selectively when they reported novel findings not yet available in peer-reviewed publications.

Studies were excluded if they focused primarily on surgical techniques, pouch function, or clinical outcomes unrelated to the gut microbiota; involved populations without an ileal pouch-anal anastomosis; or did not provide information relevant to microbiota composition, function, host–microbiota interactions, or microbiota-targeted interventions. Editorials, commentaries, and other publications without original data or substantial evidence synthesis were generally excluded, although relevant review articles were consulted to provide context and identify additional primary studies.

Additional relevant studies were identified through manual screening of the reference lists of included articles and review papers. No language-based eligibility restrictions were applied, although the vast majority of identified literature was published in English. As this was a narrative review, studies were selected based on their relevance to the review objectives and their contribution to the current understanding of microbiota-related mechanisms and therapeutic strategies in pouchitis. Given the heterogeneity of study designs, patient populations, microbiota assessment methods, and outcome measures across the included literature, a formal meta-analysis was not attempted for this review. Findings were synthesized narratively, with attention to the quality and consistency of evidence across studies.

This review did not include a formal risk-of-bias assessment or independent grading of evidence certainty. Instead, the strengths and limitations of the available evidence were evaluated narratively and interpreted in the context of existing systematic reviews, meta-analyses, and clinical practice guidelines where available. Limitations of the evidence base, including small sample sizes, heterogeneous study designs, lack of validated outcome measures for pouchitis, and the absence of standardized microbiota assessment methods, are acknowledged throughout the review.

## 3. Microbiota Alterations in Pouchitis

### 3.1. Evidence for Dysbiosis

The ileal pouch undergoes a process of colonic metaplasia following IPAA, accompanied by a fundamental shift in microbial composition from a small-bowel-type community toward a colon-like microbiota [15,18]. This transformation is driven by the altered luminal environment of the pouch, which, through fecal stasis, changes in pH, oxygen tension, and nutrient availability, selecting obligate anaerobes and other colonic commensals over the facultative anaerobes that normally predominate in the terminal ileum [18,19]. This adaptive colonization creates a microenvironment that, in genetically susceptible individuals, predisposes to dysbiosis and inflammation [11,12].

Current evidence suggests that primary pouchitis results from a dysregulated host immune response to microbial imbalance within the pouch lumen among susceptible individuals, rather than from a clearly identifiable infectious organism [11]. This concept is supported by several observations: (a) pouchitis occurs almost exclusively in UC patients and more rarely in those with familial adenomatous polyposis (FAP) who undergo the same surgical procedure, implicating disease-specific host–microbiota interactions; (b) acute pouchitis responds to antibiotic therapy in the majority of cases, suggesting a microbial driver; and (c) select probiotics can prevent pouchitis and maintain remission, further supporting the microbiota as a therapeutic target [12,15].

Importantly, the non-inflamed pouch already exhibits a dysbiotic microbial profile. Dubinsky et al. demonstrated through shotgun metagenomic analysis of 208 fecal samples from 69 pouch patients that the normal (non-inflamed) pouch microbiome resembles Crohn’s disease more than healthy controls or UC, suggesting that the pouch microenvironment is inherently predisposed to microbial imbalance even before clinical inflammation develops [20]. This finding has important implications for understanding the natural history of pouchitis and for identifying patients at risk for disease development. Additionally, a systematic review by Segal et al. summarized the available evidence on ileoanal pouch microbiota in health and disease and concluded that no specific bacterial strain was identified as pathogenic [21]. However, dysbiosis characterized by decreased microbial diversity in UC-IPAA patients may, in genetically predisposed subjects, lead to aberrant mucosal immune regulation triggering an inflammatory process [21]. Nevertheless, whether the inflammatory process causes the change in the microbiota or vice versa remains an unsolved question. However, studies showing that certain microbiota patterns can predict the development of pouchitis before inflammation occurs suggest that microbial changes may precede and contribute to disease onset [21,22].

### 3.2. Changes in Microbial Diversity

**Figure 1:**
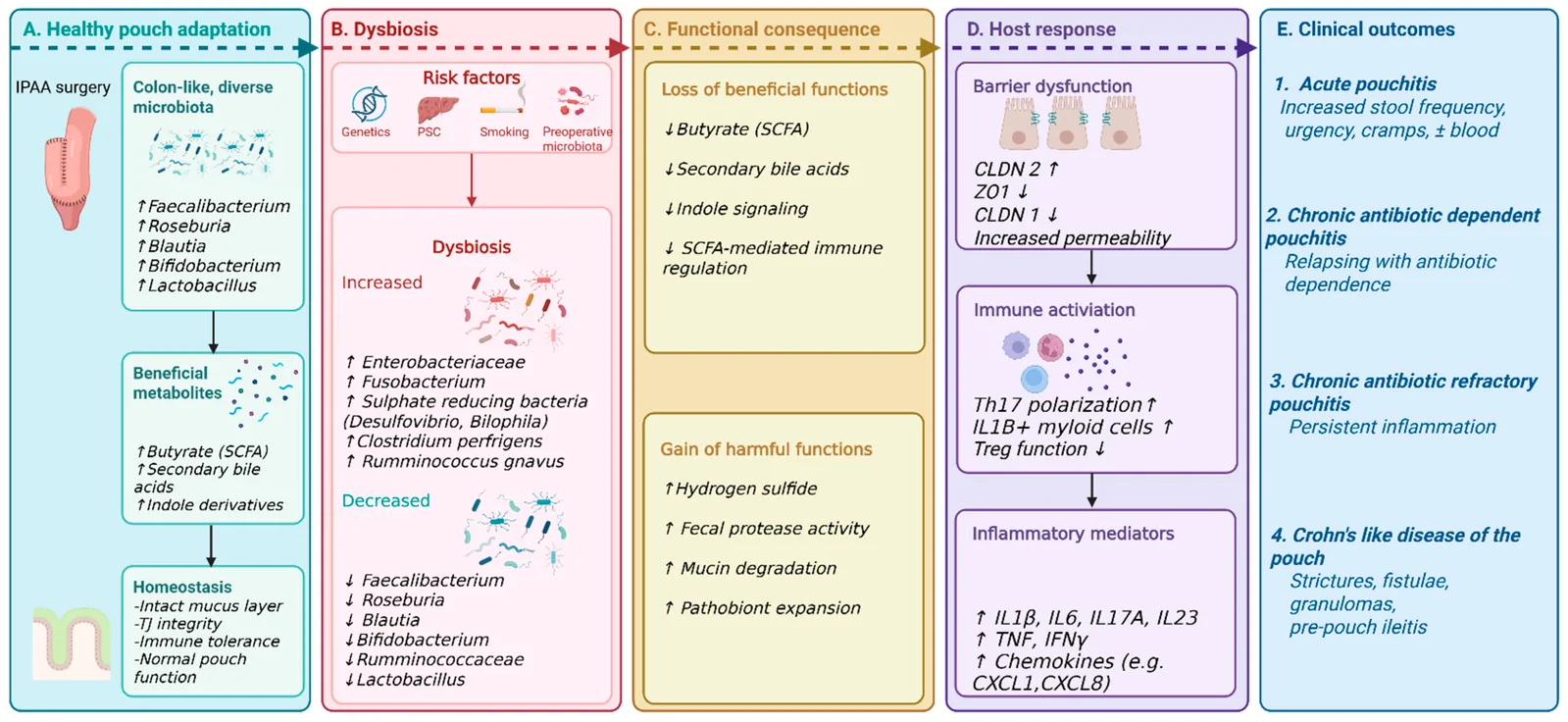
Proposed microbiota-driven mechanisms in pouchitis. Following IPAA, the ileal pouch develops a more colon-like microbiota that supports production of beneficial metabolites, including short-chain fatty acids, secondary bile acids, and indole derivatives, thereby maintaining epithelial barrier function and immune homeostasis. In susceptible individuals, dysbiosis is characterized by expansion of pathobionts and depletion of beneficial commensals, resulting in loss of protective microbial functions and increased production of potentially harmful metabolites. These alterations are associated with barrier dysfunction, immune activation, and inflammatory mediator production, ultimately contributing to acute pouchitis, chronic antibiotic-dependent pouchitis, chronic antibiotic-refractory pouchitis, and Crohn’s-like disease of the pouch. Evidence for short-chain fatty acid and secondary bile acid alterations is strongest in pouchitis, whereas some pathways, including indole signalling, are inferred largely from IBD studies. Abbreviations: IPAA, ileal pouch-anal anastomosis; SCFA, short-chain fatty acid; TNF, tumour necrosis factor; IL, interleukin; Treg, regulatory T cell; Th17, T helper 17 cell.

A hallmark of pouchitis is reduced microbial diversity (Figure 1), a finding that has been consistently demonstrated across studies using both culture-based and culture-independent molecular techniques [18,21]. Several studies have also reported substantial reductions in bacterial density within the pouch microbiota. Bacterial density in pouch effluent from UC patients is approximately 65% lower than that observed in fecal samples from healthy controls. Moreover, UC patients with a history of pouchitis exhibit a further 35% reduction in bacterial density compared with UC and FAP patients without current or previous pouchitis [23]. Using 16S rRNA amplicon sequencing, Kousgaard et al. evaluated 38 participants, including 11 patients with normally functioning pouches, 9 with chronic pouchitis, 6 with FAP, and 12 healthy controls. Patients with chronic pouchitis demonstrated significantly lower microbial diversity and richness than both patients with normal pouch function and healthy individuals [24]. Interestingly, no significant difference was found between patients with FAP and chronic pouchitis in terms of diversity or richness, suggesting that the pouch environment itself, regardless of underlying diagnosis, may constrain microbial diversity [24]. In addition to reductions in diversity and richness, episodes of pouchitis are associated with a lower anaerobic-to-aerobic bacterial ratio in both mucosal biopsies and fecal samples [18]. This shift reflects the loss of obligate anaerobes, many of which are protective butyrate producers, and the relative expansion of facultative anaerobes and aerobes, including Enterobacteriaceae [18]. Kousgaard et al. also described a gradient in microbial composition extending from healthy individuals to patients with normally functioning pouches, patients with FAP, and finally those with chronic pouchitis [24]. This pattern suggests a progressive loss of microbial complexity along the continuum from health to disease. Similar observations have been reported in other forms of inflammatory bowel disease (IBD), where reduced microbial diversity is thought to diminish ecosystem resilience and functional redundancy, thereby increasing susceptibility to environmental perturbations and pathobiont expansion. Prospective studies further support the association between microbial diversity and pouch inflammation. Reshef et al. demonstrated in a cohort of 131 UC pouch patients that specific bacterial populations correlate with pouch disease phenotype and inflammatory activity, with overall microbial richness being lower in inflamed pouches [25]. Likewise, Tyler et al. reported significantly lower Shannon diversity indices in UC pouches compared FAP pouches, in normal pouches, and in Crohn’s-like disease of the pouch [26].

Together, these findings indicate that pouchitis is characterized by marked reductions in bacterial density, diversity, and richness, accompanied by a shift away from obligate anaerobic communities toward a less stable microbial ecosystem. Such alterations may reduce microbial resilience and contribute to the development and persistence of pouchitis.

### 3.3. Temporal Evolution of the Pouch Microbiota

The microbiota of the ileal pouch undergoes dynamic changes following IPAA construction, gradually transitioning from an ileal-like to a more colonic-like microbial community, although the trajectory of these changes appears to be highly individualized [18]. Within the first two months after restoration of intestinal continuity, fecal samples show an increased abundance of colon-associated anaerobes accompanied by a decline in bacteria typically enriched in the ileum [18]. Almeida et al. reported that, compared with preoperative rectal samples, pouch samples collected two months after surgery exhibited higher proportions of *Bacteroidetes* (30% vs. 20%), *Lactobacillus* (30% vs. 0%), and *Veillonella* (90% vs. 30%), together with lower abundances of *Staphylococcus* (40% vs. 70%) and *Corynebacterium* species (30% vs. 60%) [27]. These findings illustrate the early ecological adaptation that occurs within the newly formed pouch.

Evidence from staged IPAA procedures further supports this transition. At the time of ileostomy closure, the proximal bowel is predominantly colonized by facultative anaerobes such as lactobacilli, enterococci, and coliform organisms, whereas sulphate-reducing bacteria (SRB) and *Clostridium perfringens* are present at low levels [18]. Following restoration of fecal flow, the microbial ecosystem shifts toward increased representation of obligate anaerobes and reduced abundance of facultative anaerobes, ultimately acquiring features that more closely resemble the colonic microbiota [28]. This process is thought to be driven by fecal stasis and alterations in the luminal environment of the pouch. Supporting this concept [18,28], Landy et al. demonstrated that abnormalities in tight-junction protein expression, including increased expression of the pore-forming claudin 2, occur early after ileostomy closure, suggesting that barrier dysfunction develops in parallel with microbial colonization changes [29].

Notably, susceptibility to pouchitis may be determined even before pouch formation. In a prospective study, Machiels et al. collected fecal samples from 21 UC patients before colectomy and during the first year after IPAA [22]. Cluster analysis of the pre-colectomy microbiota distinguished patients who subsequently developed pouchitis from those with a normal pouch during the first year of follow-up, demonstrating that the microbial trajectory toward pouchitis may be established even before pouch construction [22]. Individuals who later developed pouchitis displayed significant enrichment of *Ruminococcus gnavus*, *Bacteroides vulgatus*, and *Clostridium perfringens*, together with depletion of the Lachnospiraceae genera *Blautia* and *Roseburia*, in patients who subsequently developed pouchitis [22]. A prediction model incorporating these five microbial markers showed excellent sensitivity (100%) and moderate specificity (63.6%) for identifying patients at risk of developing pouchitis within the first postoperative year [22]. These observations provided the first evidence that pre-existing microbiome characteristics may predict future pouch outcomes [21]. If validated in larger cohorts, such predictive signatures could inform pre-operative counselling and provide an opportunity to alter the gut microbiota prophylactically to prevent future complications [21].

### 3.4. Taxonomic Shifts

Pouchitis is characterized by consistent taxonomic shifts that have been replicated across multiple studies using diverse methodologies. These changes can be categorized into enrichment of putatively pathogenic taxa and depletion of putatively protective taxa (Table 1). Importantly, these compositional alterations are not merely descriptive but are also associated with markers of disease activity. Reshef et al. found that increased proportions of Fusobacteriaceae correlated with greater disease activity and higher C-reactive protein (CRP) levels in UC patients with ileal pouches, whereas *Faecalibacterium* abundance was reduced in patients with pouchitis compared with controls and was negatively correlated with CRP levels, supporting a relationship between microbial dysbiosis and intestinal inflammation [25].

Although these microbial signatures have been consistently identified, their relative abundance may vary according to the sampling compartment. Segal et al. highlighted that both fecal samples and mucosal biopsies have been used to characterize the pouch microbiota, with broadly consistent findings across compartments [21]. Nevertheless, several taxa show differential abundance between mucosal and luminal samples. For example, *Lactobacillus* spp. showed a 5:1 mucosal-to-stool ratio, and *Streptococcus* spp. showed a similar 5:1 mucosal-to-stool ratio, while Bacteroidetes comprised 20% vs. 71% of total composition in mucosal vs. stool samples [25,30]. These compartmental differences underscore the importance of standardizing sampling strategies across studies.

Further evidence for disease-associated microbial signatures comes from comparisons between UC and FAP pouches. UC pouches show increased abundance of Alcaligenaceae, *Clostridium* spp., Comamonadaceae, *Eubacterium* spp., Lachnospiraceae, Moraxellaceae, *Prevotella* spp., *Roseburia* spp., sulphate-reducing bacteria, and *Veillonella* spp., while FAP pouches are enriched in Bacteroidaceae, *Akkermansia* spp., *B. vulgatus*, *Lactobacillus* spp., Prevotellaceae, Ruminococcaceae, *F. prausnitzii*, and *Streptococcus* spp. [18] An important distinction between UC and FAP pouches is the higher abundance of sulphate-reducing bacteria observed in UC [19,31]. This has been hypothesized to result from colonic metaplasia, which increases sulfomucin availability and thereby provides a favorable substrate for SRB proliferation [19,31].

The direction of these taxonomic differences should be interpreted with caution, as findings for individual genera such as *Roseburia* are not consistent across studies; apparent enrichment in UC versus FAP pouches contrasts with the reduction of *Roseburia* observed when pouches become inflamed and in active UC, reflecting differences in comparator group (FAP pouch vs. non-inflamed pouch vs. healthy control), sampling site, and sequencing methodology rather than a true reversal of its function.

### 3.5. Functional and Metabolic Alterations

Beyond compositional changes, metagenomic and metabolomic analyses have revealed that pouchitis is associated with profound functional alterations in the microbial community. Using shotgun metagenomics, Dubinsky et al. analyzed 208 fecal samples from 69 pouch patients and compared them to publicly available metagenomes from patients with CD (*n* = 88), UC (*n* = 76), and healthy controls (*n* = 56) [20]. Patients with pouchitis exhibited the most pronounced alterations in microbial composition and functional capacity, including changes in bacterial species, enzymatic activity, and metabolic pathways, which were associated with inflammatory activity [20]. In particular, pathways involved in the production of butyrate and secondary bile acids were reduced across inflammatory bowel disease phenotypes, with the greatest depletion observed in pouchitis [20]. In contrast, metabolic pathways related to amino acid synthesis and the breakdown of aromatic compounds and carbohydrates, largely attributable to members of the Enterobacteriaceae family, were enriched in both pouchitis and CD but not in UC [20]. The study also identified a marked expansion of *Ruminococcus gnavus* strains carrying genes encoding mucin-degrading glycoside hydrolases, supporting a potential role for mucus layer degradation in epithelial barrier impairment [20]. Furthermore, machine learning models based on microbial species and gene profiles successfully differentiated patients with pouchitis from those with a healthy pouch, highlighting the potential utility of microbial biomarkers for disease prediction and classification [20].

Among the functional microbial alterations described in pouchitis, the strongest disease-specific evidence relates to disturbances in short-chain fatty acid (SCFA) production, hydrogen sulphide metabolism, and secondary bile acid transformation [23]. Reduced fecal concentrations and in vitro production of SCFAs, particularly butyrate, have been consistently observed in pouchitis [23]. As the adapted pouch epithelium relies heavily on butyrate as an energy source, reductions in its availability may adversely affect epithelial barrier integrity, anti-inflammatory signaling pathways, and mechanisms involved in hydrogen sulphide detoxification. Although SCFAs generally exert anti-inflammatory effects [32], excessively high concentrations have been shown to damage the intestinal epithelium in experimental settings [23]. In parallel, elevated production of hydrogen sulphide has been linked to both active and recent pouch inflammation [23]. Sulphate-reducing bacteria including *Desulfovibrio* and *Bilophila* appear more abundantly in UC-associated pouches and in patients with active pouchitis than in FAP pouches [23]. Hydrogen sulphide can impair epithelial energy metabolism by inhibiting butyrate oxidation, thereby limiting fuel availability for intestinal epithelial cells and potentially contributing to mucosal injury [18,23]. Alterations in bile acid metabolism also appear relevant. Concentrations of secondary bile acids such as deoxycholic acid and lithocholic acid are reduced in UC pouch patients compared with those with FAP [23]. Because members of the Lachnospiraceae and Ruminococcaceae families are principally responsible for converting primary into secondary bile acids, depletion of these taxa may explain this metabolic deficit. Although secondary bile acids have demonstrated anti-inflammatory properties in experimental colitis models, excessive levels may exert toxic, pro-inflammatory, and carcinogenic effects [23].

Additional metabolic pathways may contribute to pouch inflammation, although most available evidence originates from broader IBD research rather than pouchitis-specific studies. Intestinal microorganisms convert dietary tryptophan into several indole derivatives, including indole-3-ethanol, indole-3-pyruvate, and indole-3-aldehyde, which activate the aryl hydrocarbon receptor (AhR) on intestinal epithelial cells. Activation of this pathway supports epithelial barrier function through maintenance of tight-junction integrity and cytoskeletal regulation [33]. In the setting of IBD-associated dysbiosis, production of these beneficial indole metabolites is diminished, potentially contributing to impaired barrier function [34,35]. Concurrently, inflammatory conditions promote activation of the kynurenine pathway, which diverts tryptophan metabolism away from serotonin and indole production and generates metabolites with immunoregulatory properties [36]. Although direct evidence linking tryptophan metabolism, AhR signaling, or kynurenine pathway activity to pouchitis remains scarce, depletion of microbial groups that contain indole-producing organisms, including Lachnospiraceae and *Lactobacillus* species, suggests that these pathways may play a role in disease pathogenesis and therefore warrant further investigation.

### 3.6. Beyond Bacteria: Mycobiome and Virome

While the bacterial microbiota has been the primary focus of pouchitis research, emerging evidence implicates the mycobiome (fungal community) as an additional contributor to pouch inflammation. Zhu et al. observed reduced fungal alpha diversity and increased *Saccharomyces* abundance in patients with pouchitis compared with those with normal pouches [37]. Similarly, experimentally induced fungal dysbiosis in an IPAA rat model exacerbated pouchitis and was accompanied by shifts in fungal composition, including reduced Kazachstania and increased Polythrincium and Saccharomyces, together with more severe inflammatory and clinical manifestations [37]. Blocking commensal fungal recognition through dectin-1 also increased the severity of pouchitis, suggesting that host–fungal interactions play a protective role in maintaining pouch homeostasis [37].

In a recent review focusing on the gut virome and mycobiome in IBD, Houshyar et al. emphasized that characterization of these microbial communities is complicated by substantial interindividual variability, sequencing-related limitations, contamination, and incomplete reference databases [38]. Current evidence suggests that fungal and viral dysbiosis may contribute to IBD pathogenesis, with reported alterations including expansion of *Candida* species, depletion of *Saccharomyces*, increased abundance of Caudoviricetes bacteriophages, and detection of viruses such as cytomegalovirus and Epstein–Barr virus within inflamed intestinal tissue [38]. These microbial shifts may impair epithelial barrier function, influence immune responses, and participate in complex cross-kingdom interactions that promote inflammation. Whether similar mechanisms operate within the ileal pouch environment remains uncertain. Nevertheless, the recognition that pouchitis pathogenesis may extend beyond the bacterial microbiome to encompass fungal and viral communities underscores the need for multi-kingdom microbiota profiling in future studies. Cross-kingdom interactions, including bacteriophage-mediated modulation of bacterial populations and fungal-bacterial metabolic cross-talk, represent an underexplored frontier that may reveal new therapeutic targets.

### 3.7. Secondary Pouchitis and Other Causes of Pouch Inflammation

In contrast to primary pouchitis, which is thought to arise from an aberrant immune response to the pouch microbiota in genetically susceptible individuals, secondary pouchitis results from identifiable infectious, inflammatory, pharmacological, or mechanical causes and should be considered in the differential diagnosis of pouch dysfunction.

While primary pouchitis is believed to result from dysbiosis without identifiable pathogens, secondary pouchitis can be triggered by specific infectious agents. *Clostridioides difficile* infection (CDI) of the ileal pouch has been recognized in approximately 10% of symptomatic patients seen at tertiary referral centers for pouch dysfunction [11,12]. Risk factors for CDI-associated pouchitis include male gender, recent hospitalization, and pre-surgery antibiotic use [18]. Shore et al. reported a CDI prevalence of 9.1% (18/198) in patients with chronic antibiotic-dependent pouchitis or Crohn’s-like disease of the pouch, with preoperative history of CDI being the greatest risk factor for postoperative CDI (67% vs. 6%; *p* < 0.001) [39].

Other infectious causes should also be considered. Furthermore, cytomegalovirus (CMV) can cause chronic pouchitis, and patients with chronic antibiotic-refractory pouchitis should be evaluated for CMV infection with serology or pouchoscopy and biopsy [11,12]. CMV-associated pouchitis can be treated with ganciclovir or valganciclovir.

In addition to specific pathogens, alterations in the pouch microbial ecosystem may complicate disease management. For example, extended-spectrum beta-lactamase (ESBL)-producing bacteria are commonly found (33%) in patients with recurrent or refractory pouchitis or prepouch ileitis, highlighting the potential role of antimicrobial resistance in shaping the pouch microbial ecosystem and complicating antibiotic therapy [11]. Other gastrointestinal luminal bacterial pathogens, such as *C. perfringens* and *Klebsiella pneumoniae*, as well as commensal bacteria such as *R. gnavus*, can be found in patients with pouchitis and may contribute to disease perpetuation.

Non-infectious secondary causes of pouch inflammation should also be excluded, such as NSAID-induced pouchitis, radiation pouchitis, and pouch outlet obstruction, the modification of which often helps improve or resolve pouchitis [40].

## 4. Mechanisms Linking Dysbiosis to Pouch Inflammation

### 4.1. Genetic Susceptibility

The observation that pouchitis occurs almost exclusively in UC patients, and only rarely in FAP patients who undergo the same surgical procedure, strongly implicates disease-specific genetic susceptibility in shaping host–microbiota interactions [15]. Although most genetic association studies in pouchitis are small and underpowered, several candidate loci have been identified that may influence the host immune response to pouch microbiota.

The strongest genetic association identified to date links chronic pouchitis in Caucasian populations with the NOD2 frameshift variant rs2066847 (NOD2 insC; 1007fsCins) [41]. NOD2 recognizes muramyl dipeptide (MDP) of Gram-positive and Gram-negative bacteria and stimulates the enteric immune system; reduction or loss of function of the NOD2 protein may therefore result in an altered immune response to the intestinal microbiota [21]. In a multicentre cohort of pouch patients, possession of the NOD2 insC variant was associated with a more than threefold increased risk of chronic pouchitis [21]. These findings were subsequently validated by Tyler et al. in a larger North American cohort, where NOD2 insC emerged as the genetic variant most strongly associated with adverse pouch outcomes, including both chronic pouchitis and Crohn’s-like disease of the pouch [41]. Of note, NOD2 polymorphisms are classically linked to Crohn’s disease rather than UC, raising the possibility that UC patients who develop chronic pouch inflammation may possess a Crohn’s disease-like genetic susceptibility that becomes apparent within the pouch environment [21]. Furthermore, a composite model integrating clinical (smoking status, family history of IBD), serological (ASCA IgG, pANCA, anti-CBir1), and genetic factors significantly improved risk prediction for chronic pouchitis and Crohn’s-like disease of the pouch [41].

Additional genetic associations have been reported, including variants in TNFSF15 (associated with severe pouchitis), IL-1 receptor antagonist (IL-1RN*2), ATG16L1, JAK2, TLR9, CD14, NOX3, DAGLB, and NCF4, though most require confirmation in larger cohorts [21]. The involvement of autophagy-related genes (ATG16L1) is of particular interest given the role of autophagy in bacterial clearance and the interaction between NOD2 and autophagy pathways in maintaining intestinal homeostasis [21].

### 4.2. Barrier Dysfunction

The intestinal barrier of the ileal pouch may be particularly susceptible to injury as a consequence of the metabolic adaptations that accompany colonic metaplasia. The pouch mucosa transitions from glutamine-dependent to butyrate-dependent energy metabolism [18]. Although enterocytes normally utilize both glutamine and butyrate as major energy substrates, glutamine oxidation declines following pouch construction whereas butyrate oxidation is maintained, indicating that the adapted pouch epithelium increasingly relies on butyrate to meet its metabolic requirements [42]. This metabolic reprogramming may increase vulnerability to butyrate deficiency, particularly when key butyrate-producing bacteria, including members of *Clostridium* clusters IV and XIVa such as *Faecalibacterium prausnitzii*, *Roseburia* spp., and *Eubacterium rectale*, are depleted [43]. Although *Roseburia* has been reported as relatively more abundant in UC than FAP pouches, the more consistent and clinically relevant observation is its depletion, together with other butyrogenic Clostridium cluster XIVa/IV members, in inflamed pouches and in active UC, indicating that barrier vulnerability is driven by loss of aggregate butyrogenic capacity rather than the behaviour of any single genus. Notably, impaired butyrate metabolism has been documented in the terminal ileum of patients with UC even before IPAA, suggesting that susceptibility to barrier dysfunction may predate pouch creation [18].

Several mechanisms have been implicated in the disruption of epithelial integrity in pouchitis. First, Landy et al. demonstrated that expression of claudin-2, a pore-forming tight-junction protein, increases shortly after ileostomy closure, indicating that alterations in epithelial permeability occur early in disease development [29]. In contrast, established pouchitis is associated with reduced expression of the tight-junction proteins ZO-1 and claudin-1, consistent with impaired barrier integrity [29]. These findings were supported by experimental work in a DSS-induced rat model of pouchitis, where reduced occludin expression and elevated plasma D-lactate levels, a marker of intestinal permeability, were observed [44].

A second mechanism of barrier dysfunction in pouchitis involves microbially derived fecal proteases [45]. Patients with pouchitis exhibit markedly increased fecal protease activity compared with both healthy controls and individuals with non-inflamed pouches [45]. Among the taxa associated with this phenomenon, *Haemophilus* showed a strong positive correlation with protease activity [45]. Additional functional experiments demonstrated that faecal supernatants from pouchitis patients activated protease-activated receptor 2 (PAR2) on Caco-2 monolayers, disrupted tight-junction proteins, and increased epithelial permeability [45]. Genetic inactivation of PAR2 abolished these effects, while activated PAR2 was detected in pouch biopsies from patients with pouchitis but not in those with normal pouch function [45]. Pouch biopsies from pouchitis patients, but not from normal pouch patients, displayed PAR2 activation, suggesting that protease-producing bacteria may initiate or propagate pouch inflammation through this mechanism [45].

A third pathway involves degradation of the protective mucus layer. Organisms such as *Ruminococcus gnavus* and *Akkermansia muciniphila* possess mucinolytic capabilities that may compromise mucosal defenses and facilitate increased epithelial permeability [20,23]. Metagenomic studies have shown enrichment of *R. gnavus* strains carrying genes encoding mucin-degrading glycoside hydrolases in pouchitis, supporting a mechanistic link between microbial mucus degradation and barrier dysfunction [20].

Finally, hydrogen sulphide represents another potential mediator of epithelial injury [46,47]. Sulphate-reducing bacteria, which are enriched in UC-associated pouches, generate hydrogen sulphide that can interfere with epithelial energy metabolism by inhibiting butyrate oxidation [46,47]. Experimental studies have demonstrated this effect directly, and even non-inflamed UC pouches exhibit increased abundance of hydrogen sulphide-producing bacteria, suggesting an underlying predisposition to epithelial vulnerability [48]. Consistent with this concept, reduced levels of SCFAs have been described during pouchitis compared to the uninflamed ileal pouch, whereas successful antibiotic treatment has been shown to restore SCFA levels toward normal [42,49].

### 4.3. Microbial Colonization, Adherence, and Translocation

Beyond the compositional and metabolic dysbiosis described above, an emerging body of work addresses how pouch microbes physically and immunologically engage the host, although much of this evidence remains correlative and some is extrapolated from Crohn’s disease. Beyond the compositional and metabolic dysbiosis described above, an emerging body of work addresses how pouch microbes physically and immunologically engage the host, although much of this evidence remains correlative and some is extrapolated from Crohn’s disease [50]. Colonization of the pouch is shaped by the metaplastic niche itself as the colonic metaplasia increases mucosal sulfomucin content, providing substrate that favours colonization by sulphate-reducing bacteria, which are more abundant in UC than in the rarely inflamed FAP pouch [50]. Among these organisms, *Bilophila wadsworthia* not only generates hydrogen sulphide but also adheres to intestinal epithelial cells and degrades the protective mucus layer, coupling metabolite production to direct barrier injury [51].

Direct evidence of bacterial adherence to and invasion of the pouch epithelium is very limited. However, the Enterobacteriaceae consistently enriched in pouchitis include the adherent–invasive Escherichia coli (AIEC) pathotype, which binds inflammation-induced CEACAM6 via the FimH adhesin, invades epithelial cells, survives within macrophages, and induces TNF-α and IL-17 [52,53]. Whether an AIEC-type adherence–invasion phenotype operates in the pouch has not been directly demonstrated and represents a testable hypothesis [52,53].

Impaired barrier integrity may in turn permit systemic uptake of microbial constituents. Increased mucosal permeability to microbiota has been demonstrated in the ileal pouch, and bacterial DNA has been detected in the mesenteric lymph nodes and mesenteric adipose tissue of pouch patients, which more closely resembles the mucosal than the fecal microbiome [54]. Positive lymph node bacterial DNA was significantly associated with the development of pouchitis within 12 months (8/14 vs. 0/13), suggesting that translocation of mucosa-associated bacteria may precede and predict inflammation, although this study was small (*n* = 27) and detected bacterial DNA, not viability [54].

### 4.4. Immune Dysregulation

Microbial engagement of the pouch mucosa activates both innate and adaptive immunity. Microbe-associated molecular patterns are sensed by epithelial and innate pattern-recognition receptors (Toll-like and NOD-like receptors), shaping the balance between regulatory and effector (Th1/Th17) responses [55]. In the pouch specifically, aberrant Toll-like receptor expression has been reported in inflamed and non-inflamed pouches, together with increased Paneth-cell human β-defensin-5, expansion of immature plasma cells, elevated TNF, and an imbalance between pro-inflammatory (IL-8) and regulatory (IL-10) cytokines; UC pouches exhibit higher pro-inflammatory cytokine levels than FAP pouches [55,56]. These findings indicate that innate immune dysregulation is present even before apparent inflammation and may prime the mucosa for the adaptive responses described below.

Beyond epithelial barrier dysfunction, increasing evidence suggests that aberrant immune responses play a role in the maintenance and perpetuation of pouch inflammation. single-cell transcriptomics has begun to reveal the cellular landscape of pouch inflammation. Devlin et al. identified IL1B/LYZ+ myeloid cells and FOXP3/BATF+ T cells that distinguish inflamed pouch tissues, with IL1B+ myeloid cells interacting with Bacteroidales and Clostridiales taxa and predicting nonresponsiveness to anti-integrin therapy [57]. Further evidence of immune dysregulation was provided by Cao et al., showing that Crohn’s-like disease of the pouch is characterized by Th17 polarization, TREM1+ monocyte expansion, and heightened endoplasmic reticulum stress, a potential novel diagnostic biomarker and therapeutic target [58]. These findings support the emerging concept that pouchitis is not solely a disorder of microbial dysbiosis or epithelial barrier dysfunction but rather a consequence of complex interactions between the microbiota, epithelial cells, innate immune cells, adaptive immune responses, and stromal compartments. Consistent with this, Braga-Neto et al. proposed that pouchitis may represent a transmural disease in which stromal cells and other non-immune cellular populations contribute to the perpetuation of inflammation beyond the mucosal surface [59].

## 5. Clinical Evidence Supporting a Pathogenic Role of the Microbiota

### 5.1. Antibiotic Responsiveness

The clinical responsiveness of pouchitis to antibiotic therapy constitutes one of the strongest lines of evidence supporting a pathogenic role for the microbiota. Acute pouchitis responds to antibiotics in the majority of patients, suggesting that luminal microbial factors contribute directly to disease pathogenesis rather than merely representing secondary changes associated with inflammation [14]. Assuming a spontaneous improvement rate of 40%, antibiotic therapy was associated with a 67% higher likelihood of clinical response (RR 1.67; 95% CI 1.34–2.01) [14]. Furthermore, clinical improvement often occurs rapidly following treatment initiation, supporting a direct relationship between microbial manipulation and symptom resolution [60].

Additional support for a microbial contribution comes from the observation that disease frequently recurs following antibiotic withdrawal. Barnes et al. reported recurrent pouchitis in 70% of patients within 12 months after treatment of an initial episode [61], while Dubinsky et al. observed relapse in 89% of patients within three months after completion of a four-week antibiotic course [62]. These findings suggest that antibiotic therapy may temporarily suppress a pro-inflammatory microbial state without permanently correcting the underlying ecological disturbance.

Taken together, the responsiveness of pouchitis to antibiotics and the tendency for disease recurrence after treatment strongly support a causal role for the microbiota in the initiation and maintenance of pouch inflammation.

### 5.2. Associations with Disease Severity, Recurrence and Biomarker Potential

Multiple studies have demonstrated that specific microbial signatures correlate with disease severity, phenotype, and recurrence risk, further supporting a pathogenic role for the microbiota. First of all, Reshef et al. prospectively studied 131 UC pouch patients and found that increased proportions of the *Fusobacteriaceae* family correlated with increased disease activity and CRP levels, while proportions of *Faecalibacterium* were reduced in pouchitis and negatively correlated with CRP [25]. Dubinsky et al. demonstrated that patients with pouchitis presented the highest alterations in species, metabolic pathways, and enzymes compared to normal pouches, CD, UC, and healthy controls, with these alterations correlating with intestinal inflammation [20]. Microbial feature-based classifiers could distinguish between patients with a normal pouch and those with pouchitis, identifying species and genes predictive of disease [20]. Secondly, the International Ileal Pouch Consortium has classified pouchitis into acute, chronic antibiotic-responsive, chronic antibiotic-dependent (CADP), and chronic antibiotic-refractory (CARP) phenotypes [63]. Each phenotype appears to have distinct microbial characteristics. Patients with CADP require continuous or frequently repeated antibiotic courses, suggesting persistent microbial drivers, while CARP, which affects approximately 5–10% of pouchitis patients, may involve microbial communities that are resistant to conventional antibiotic manipulation and may require advanced immunosuppressive therapies [6,11]. Advanced therapies were effective for treatment of CARP (31 cohorts; RR 1.71; 95% CI 1.28–2.56; response rate 50%; 95% CI 43–57) without significant difference between classes [14]. Third, the high recurrence rate after antibiotic cessation, 89% within 3 months in the Dubinsky et al. cohort, is in itself evidence that the underlying microbial perturbation is not corrected by antibiotics [62]. Barnes et al. found that 70% (190/271) of patients treated with antibiotics for an initial episode of pouchitis developed recurrent pouchitis within 12 months, regardless of antibiotic choice [61]. This pattern of relapse suggests that the microbial ecosystem rapidly reverts to a pro-inflammatory state after antibiotic withdrawal, with blooms of oral and disease-associated bacteria observed within days of cessation [62].

Furthermore, the demonstration that pre-colectomy microbial signatures can predict subsequent pouchitis development provides perhaps the most compelling evidence for a causal role of the microbiota. Machiels et al. prospectively showed that cluster analysis of the pre-colectomy fecal microbiota distinguished patients who subsequently developed pouchitis from those with a normal pouch during the first year of follow-up [22]. A score combining five bacterial risk factors (*R. gnavus*, *B. vulgatus*, *C. perfringens*, *Blautia*, and *Roseburia*) showed a sensitivity of 100% and specificity of 63.6% for predicting pouchitis within the first year after IPAA when at least two risk factors were present [22]. This temporal precedence of microbial changes before clinical disease onset supports a causal rather than merely associative relationship between dysbiosis and pouchitis.

Therefore, the development of non-invasive biomarkers for pouchitis diagnosis, monitoring, and risk stratification represents an important translational application of microbiota research. Fecal calprotectin (FC) has emerged as the most promising non-invasive biomarker for pouchitis. Ollech et al. demonstrated in a cross-sectional study of 156 patients (296 encounters) that median FC values were significantly lower in patients without pouchitis compared to those with pouchitis (208 vs. 550 μg/g), with FC performing better than CRP as a predictor of pouchitis [64]. An FC > 460 μg/g had >80% specificity for predicting significant endoscopic disease (PDAI endoscopic subscore ≥ 5), while FC < 125 μg/g had >80% specificity for predicting endoscopic remission [64]. Li et al. confirmed these findings, reporting a significant correlation coefficient of 0.651 between FC and PDAI score, with an FC cutoff of 579.60 μg/g for predicting pouchitis (AUC 0.938) and 143.25 μg/g for predicting high risk of pouchitis (PDAI 3–6; AUC 0.876) [65]. Most recently, Bronze et al. (2026) analyzed 163 patients from a prospectively maintained IPAA registry and found that FC distinguished between inflammatory and normal pouches with an AUC of 0.82, with an optimal threshold of approximately 167 μg/g (specificity 94%) and a cutoff of 280 μg/g for predicting severe endoscopic activity (AUC 0.82) [66]. Median FC values differed significantly across pouch phenotypes: 50.5 μg/g in normal pouches, 244 μg/g in acute pouchitis, 370.5 μg/g in chronic pouchitis, and 231.5 μg/g in Crohn’s disease-like pouch inflammation [66]. The AGA guideline notes that prior studies have demonstrated that FC is correlated with the PDAI, specifically endoscopic inflammation, and that FC levels elevate prior to a clinical diagnosis of pouchitis [6]. Lactoferrin also appears to increase prior to a diagnosis of pouchitis and correlates with endoscopic inflammation [6]. However, these biomarkers are not yet routinely used in clinical practice for pouch management [6].

Beyond conventional inflammatory markers, microbial signatures themselves hold biomarker potential. The pre-colectomy microbial risk score developed by Machiels et al. could serve as a predictive biomarker for pouchitis risk stratification [22]. The metagenomic classifiers developed by Dubinsky et al., which distinguish normal pouches from pouchitis based on species and gene profiles, represent another avenue for microbial biomarker development [20]. Fecal calprotectin levels decreased by 40% in response to antibiotics in the Dubinsky et al. cohort, suggesting that calprotectin could serve as a surrogate marker for monitoring treatment response [62]. Fecal SCFA concentrations, hydrogen sulphide levels, and secondary bile acid profiles represent additional candidate biomarkers that reflect the functional state of the pouch microbiota [23]. However, these metabolic biomarkers have not been validated in prospective clinical studies and remain research tools rather than clinical instruments.

## 6. Therapeutic Modulation of the Pouch Microbiota

**Figure 2:**
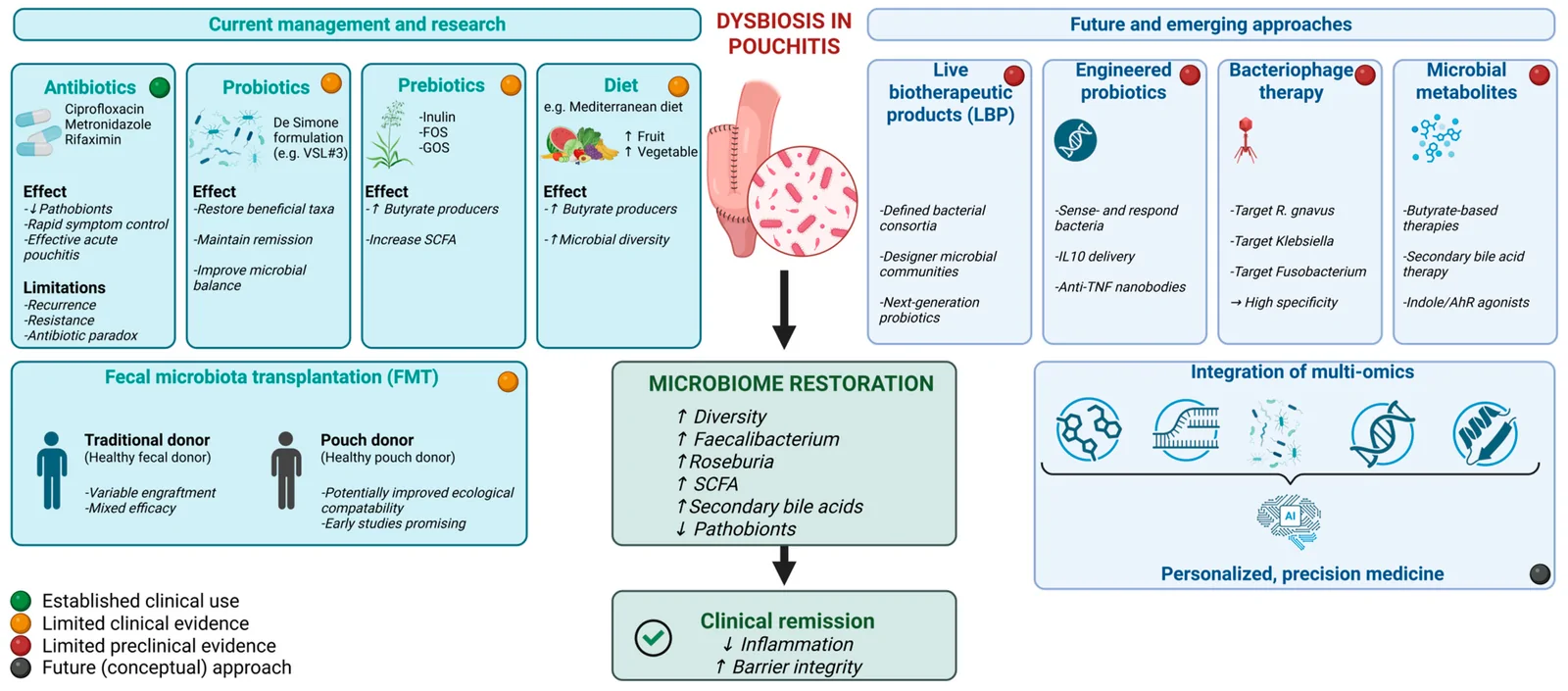
Current and emerging microbiota-targeted strategies for pouchitis. Microbiome-based interventions for pouchitis can be classified according to their stage of clinical development and supporting evidence. Current management and research approaches include antibiotics, probiotics, prebiotics, dietary interventions, and fecal microbiota transplantation (FMT). Among these, antibiotics have the strongest clinical evidence and remain the standard of care for acute pouchitis. Probiotics are supported by moderate clinical evidence, particularly for maintenance of remission, whereas prebiotics, dietary interventions, and FMT currently have limited or inconsistent clinical evidence. Future and emerging approaches, including live biotherapeutic products, engineered probiotics, bacteriophage therapy, and microbiome-derived metabolites, remain investigational and are supported primarily by preclinical studies or indirect evidence from inflammatory bowel disease and other gastrointestinal disorders, with little or no pouchitis-specific clinical evidence currently available. Integration of multi-omics technologies represents a conceptual and translational approach with potential to enable microbiome-guided precision medicine and personalized treatment strategies. Abbreviations: FMT, fecal microbiota transplantation; LBP, live biotherapeutic product; SCFA, short-chain fatty acid; TNF, tumour necrosis factor; AhR, aryl hydrocarbon receptor.

Multiple microbiome-targeted interventions have been investigated as potential approaches for the management of pouchitis (Figure 2).

### 6.1. Antibiotics

The clinical responsiveness of pouchitis to antibiotic therapy constitutes one of the strongest lines of evidence supporting a pathogenic role for the microbiota. Antibiotics remain the primary initial treatment for pouchitis, with a pooled response rate of 65% (95% CI 52–75%) across 12 cohorts in a systematic review and meta-analysis by Syal et al. [14]. With assumed spontaneous improvement rates of 40%, antibiotics were associated with a 67% higher risk of clinical response (RR 1.67; 95% CI 1.34–2.01) [14]. Most patients with pouchitis initially respond to ciprofloxacin (500–1000 mg/day) or metronidazole (750–1500 mg/day), with symptomatic improvement typically occurring within 1–2 day [60].

In the only head-to-head RCT, Shen et al. randomized 16 patients with acute pouchitis (PDAI > 7) to ciprofloxacin or metronidazole for two weeks [60]. Remission (PDAI < 7) was achieved in 100% (7/7) of the ciprofloxacin group compared to 33% (3/9) of the metronidazole group (RR 2.68; 95% CI 1.13–6.35), though the certainty of evidence was very low [60]. A larger propensity-matched retrospective study by Barnes et al. (*n* = 271) found no significant difference in the odds of early relapse or nonresponse between ciprofloxacin, metronidazole, or combination therapy, and no difference in odds of recurrent pouchitis at 12 months [61]. Ciprofloxacin may generally be better tolerated than metronidazole, which is associated with vomiting, dysgeusia, and transient peripheral neuropathy [6,67].

Alternative antibiotic strategies have also been explored. In a pilot RCT, rifaximin (400 mg three times daily) did not achieve statistically significant differences in clinical remission or response compared to placebo at 4 weeks, though the rifaximin group had a significantly larger change in PDAI from baseline (MD −2.50; 95% CI −4.91 to −0.09) [68,69]. A network meta-analysis by Poo et al. ranked rifaximin as the best antibiotic for acute pouchitis when compared to placebo, though this was based on very limited data [70]. For maintenance therapy, a case series of 51 patients treated with rifaximin (median dose 200 mg/day) reported that 65% maintained remission at 3 months, with 58% continuing at 12 months [71].

Patients with recurrent, chronic antibiotic-dependent, or chronic antibiotic-refractory pouchitis frequently require repeated or prolonged antibiotic treatment [6]. The AGA guideline notes that combination therapy (e.g., ciprofloxacin plus metronidazole) has typically been reserved for patients with incomplete response to initial treatment or recurrent episodes, though expert opinion indicates some gastroenterologists may choose combination therapy or a 4-week course for an initial episode [6].

Long-term antibiotic use is common among pouch patients. In a cohort of 205 UC patients with IPAA, Bar et al. reported that 81.5% had received antibiotics for pouchitis and that long-term antibiotic use increased from 18% at 5 years after IPAA to 42% at 20 years [72]. Although adverse event rates were low and no increase in clinically apparent resistant infections was observed, concerns regarding antimicrobial resistance and long-term ecological consequences remain [72]. Therefore, the interest in microbiota-directed alternatives has increased like probiotics and diet [62].

### 6.2. Probiotics

Probiotics represent the most extensively studied microbiota-directed therapy for pouchitis, with the strongest evidence supporting the use of the 8-strain De Simone formulation (formerly known as VSL#3) for both primary and secondary prevention [73].

Meta-analysis of four RCTs demonstrated that probiotics were associated with an 82% lower risk of developing pouchitis compared to placebo (RR 0.18; 95% CI 0.05–0.62) [6,14]. The AGA 2020 guidelines on probiotics noted that two studies of the 8-strain formulation showed that patients receiving probiotics were more likely to have zero acute pouchitis episodes over 12 months compared to placebo or no treatment (RR for no episodes 1.29; 95% CI 1.03–1.61), though with very low certainty of evidence [74]. Despite this signal, the AGA 2024 pouchitis guideline recommended neither in favor of nor against probiotics for primary prevention, citing uncertainty about the benefit, unclear duration of prophylaxis (potentially lifelong), limited insurance coverage, and concern about exacerbating inequities [6]. A subset of patients at high risk of pouchitis, such as those with primary sclerosing cholangitis, may consider using probiotics for primary prevention, though this strategy has not been specifically studied in high-risk populations [6].

The evidence for secondary prevention and maintenance of remission is more robust. A pooled analysis of two RCTs (*n* = 76) showed that 85% (34/40) of patients receiving the De Simone formulation maintained remission at 9–12 months compared to 3% (1/36) of placebo recipients (RR 18.50; 95% CI 3.86–88.56; moderate certainty evidence) [69]. In the Gionchetti et al. trial, the relapse rate at 9 months was 15% (3/20) for probiotic therapy compared to 100% (20/20) for placebo [73]. In the Mimura et al. trial, remission at 1 year was maintained in 85% (17/20) of the De Simone formulation group and 6% (1/16) of the placebo group [75]. A meta-analysis of three RCTs confirmed that probiotics were associated with an 83% lower risk of relapse over 12 months (RR 0.17; 95% CI 0.09–0.34) [6]. The AGA 2024 pouchitis guideline conditionally suggests using probiotics for preventing recurrent pouchitis in patients with antibiotic-responsive disease (conditional recommendation, low certainty of evidence) [6]. ECCO guidelines recognize that there is limited and conflicting evidence [76]. Nevertheless, real-world effectiveness may be lower than trial results suggest. A postmarketing study of 31 patients with antibiotic-dependent pouchitis reported that at 8-month follow-up, only 6 patients remained on the De Simone formulation, with 25 discontinuing due to recurrence of symptoms or adverse effects [77]. The AGA guideline panel noted that clinical practice does not mirror the high efficacy observed in clinical trials, raising concern for publication bias [6].

The evidence for probiotics in treating active pouchitis is weaker. Three studies using different probiotic formulations showed a pooled clinical response rate of 52% (47/84; 95% CI 27–76%), with an estimated 40% higher likelihood of response compared to no treatment (RR 1.40; 95% CI 1.12–1.86) [6]. The single RCT of *Lactobacillus rhamnosus* GG for treatment showed only 1/10 patients responding compared to 0/10 with placebo [78]. The AGA guideline panel noted that in collective clinical experience, probiotics have not been very effective for the treatment of pouchitis, and there is potential that delaying therapy or using probiotics when they are not as effective as antibiotics may significantly impact quality of life [6].

Also other probiotic formulations have been studied. Single trials of *L. rhamnosus* ATCC 53103, *B. longum* Reuter ATCC BAA-999, and *C. butyricum* CBM 588 did not show clear evidence of benefit for treatment or prevention of pouchitis, though sample sizes were extremely small [74]. However, a recent retrospective pediatric study by Koike et al. found that the incidence of acute pouchitis was significantly lower in the *C. butyricum* MIYAIRI 588 (CBM) group than in the non-CBM group (10.5% vs. 58.3%), and even among patients taking any probiotics postoperatively, the CBM group had significantly lower incidence of both acute and chronic pouchitis than the “other probiotics” group [74,79]. These findings, while retrospective, suggest that CBM may deserve further evaluation in prospective trials.

Probiotics exert their effects through multiple mechanisms relevant to pouchitis pathogenesis, including suppression of pathogen growth by releasing antimicrobial mediators (lactic acid, hydrogen peroxide, acetic acid, bacteriocins), immunomodulation through depression of pro-inflammatory cytokine secretion and induction of anti-inflammatory cytokines (particularly IL-10), enhancement of barrier activity, and suppression of T-cell proliferation [80]. The De Simone formulation has been shown to increase fecal concentrations of *Lactobacillus*, *Bifidobacterium*, and *Streptococcus thermophilus* in the pouch and to modulate mucosal immune responses [81].

A meta-analysis by Estevinho et al. confirmed that probiotics decreased the odds of recurrence in relapsing pouchitis (OR 0.03; 95% CI 0.00–0.25), with multi-strain formulations appearing superior to single-strain formulations in achieving remission and preventing recurrence [82]. The Emile et al. meta-analysis of 7 trials on probiotics revealed significantly lower odds of pouchitis with probiotics (RR 0.26; 95% CI 0.16–0.42; *I*^2^ = 20%) and similar odds of adverse effects to placebo [83].

### 6.3. Prebiotics and Synbiotics

Prebiotics are non-digestible food components that selectively stimulate the growth or activity of beneficial gut bacteria. Their theoretical appeal in pouchitis lies in their potential to increase butyrate production, promote saccharolytic fermentation, and restore microbial diversity [84].

Among the different prebiotic compounds, inulin has generated the most direct clinical evidence in pouchitis. A 3-week randomized, placebo-controlled, crossover trial in 20 pouch patients (17 UC and 3 FAP) without active pouchitis demonstrated that supplementation with a large inulin dosage (24 g/day) had no effect on patients’ symptoms (clinical PDAI subscore) but significantly reduced the endoscopic and histological PDAI subscores [85]. This dissociation between symptomatic and objective inflammatory endpoints is noteworthy and suggests that prebiotics may modulate subclinical inflammation without producing clinically perceptible benefit in the short term.

Beyond inulin, other oligosaccharide-based prebiotics have been proposed as potential microbiota-modulating strategies. Fructo-oligosaccharides (FOS) and galacto-oligosaccharides (GOS) have been shown to promote the growth of *Bifidobacterium* and *Lactobacillus* species and to increase SCFA production in the colon [80]. In the context of pouchitis, FOS and GOS could theoretically promote cross-feeding relationships that increase butyrate production by Clostridium clusters XIVa and IV [23]. However, no clinical trials have specifically evaluated FOS or GOS supplementation in pouchitis patients. Similarly, lactulose and lactosucrose have been found to induce the growth of certain types of host microflora, resulting in enriched enteric function, and have been reported to exert anti-inflammatory effects by inhibiting the expression of TNF-α-related cytokines while augmenting IL-10 levels [80]. Nevertheless, their potential role in pouchitis has not been specifically studied.

Taken together, the available evidence suggests that prebiotics may represent a promising adjunctive approach for modulating pouch microbial ecology and inflammatory activity, although clinical data remain limited. In this context, the combination of probiotics with prebiotics (synbiotics) has theoretical advantages over either component alone, as the prebiotic substrate may enhance the survival and metabolic activity of the co-administered probiotic organisms. However, no clinical trials of synbiotics in pouchitis have been published. The concept remains attractive given the complementary mechanisms of action, and future trials should evaluate whether synbiotic formulations provide additive or synergistic benefit compared to probiotics alone.

### 6.4. Dietary Interventions

Dietary interventions represent an appealing therapeutic strategy for pouchitis given the interplay between diet, the pouch microbiota, and mucosal inflammation. However, the evidence base remains limited, largely consisting of observational studies and small feasibility trials, with no dietary trials on primary or secondary prophylaxis of pouchitis having been performed to date [23]. A recent dedicated review comprehensively summarized the current evidence, challenges, and opportunities for dietary modulation of the microbiota in chronic pouchitis, highlighting both the promise of nutrition-based interventions and the substantial gaps that remain in the evidence base [86].

Some of the most informative data originate from observational work conducted at the Comprehensive Pouch Clinic of the Tel Aviv Medical Centre. In these studies, individuals without a history of pouchitis reported substantially greater consumption of fruits and vegetables than patients with recurrent acute or chronic pouchitis [87]. They also had higher intakes of fat-soluble antioxidants as well as vitamins A and C [87]. In a larger cohort, low fruit consumption was associated with a significantly increased risk of developing pouchitis during follow-up [88]. Moreover, greater fruit and vegetable intake correlated with increased microbial diversity and enrichment of butyrate-producing taxa, including *Faecalibacterium*, *Lachnospira*, and members of the *Ruminococcaceae* family [88]. Similarly, stronger adherence to a Mediterranean dietary pattern was associated with a lower incidence of pouchitis, and higher Mediterranean diet scores were linked to increased intake of dietary fibre, vitamins, minerals, and antioxidants [89].

Interventional dietary studies have produced less consistent findings. In a short-term crossover trial, both the Mediterranean diet and the Specific Carbohydrate Diet (SCD) resulted in modest improvements in clinical disease activity, although neither intervention significantly affected CRP or fecal calprotectin concentrations [90]. Despite the limited clinical impact, each diet was associated with rapid but distinct alterations in microbial composition [90]. In a small case series involving patients with chronic pouchitis, an exclusive elemental diet substantially reduced stool frequency and improved clinical symptoms, yet failed to induce measurable endoscopic or histological improvement [91]. Likewise, a recent study evaluating a fibre-adapted Crohn’s Disease Exclusion Diet (CDED) demonstrated a significant reduction in modified PDAI scores among patients with active pouchitis. However, nearly half of participants discontinued the intervention because of symptom exacerbation and/or bowel obstruction [92]. These findings suggest that dietary therapies may provide benefit in selected patients but also highlight the potential limitations and risks of restrictive dietary approaches, reinforcing the importance of individualized nutritional management.

More recently, Ardalan et al. proposed the 5URE (5 strategies for a sulphide-reducing) diet as a pathogenically targeted dietary approach to prevent pouchitis [23]. This strategy addresses five pathogenic factors through dietary manipulation. The first includes reducing hydrogen sulphide production by limiting animal protein intake (sulphur-amino acids) and suppressing protein fermentation by promoting carbohydrate fermentation. Second, normalizing butyrate delivery to the pouch mucosa by delivering readily fermented carbohydrates and targeting fibres whose fermentation favours butyrate production. Third, reducing food preservatives as substrates for sulphate-reducing bacteria and nitric oxide production. Fourth, increasing intake of anti-inflammatory dietary components (omega-3 fatty acids, polyphenols). Lastly, optimizing micronutrient intake (zinc, vitamin D) for barrier function and immune regulation.

The related 4-SURE (4-strategies for sulphide-reducing) diet, with increased intake of fermentable fibers, restriction of total and sulfur-containing proteins, and avoidance of specific food additives, was evaluated in an open-label feasibility study in 28 adults with mild to moderately active UC (not specifically pouchitis) [93]. The diet was well tolerated (VAS 19 mm on a 100-mm scale), with 95% frequently or always adherent [93]. Clinical response occurred in 46% and endoscopic improvement in 36%, with fecal SCFA excretion significantly increasing by 69% and the proportion of branched-chain fatty acids (a marker of protein fermentation) significantly decreasing by 27% [93]. A diet modelled on the 4-SURE approach (called Monash Pouch Diet) had promising effects in its first open evaluation, though increments of improvement were small [94].

The current AGA Clinical Practice Update on Diet and Nutritional Therapies in IBD, mainly based on expert opinion, recommends that all patients with IBD follow a Mediterranean diet rich in fresh fruits and vegetables, monounsaturated fats, complex carbohydrates, and lean proteins and low in ultraprocessed foods, added sugar, and salt [95]. For patients with an ileal pouch, following a Mediterranean-style diet (adapted in fibre content for tolerability) for prevention and treatment of pouchitis, avoiding foods rich in FODMAPs to reduce the volume of pouch effluent, and adjusting meal timing to minimize nocturnal symptoms seems reasonable, though evidence is limited [96]. Ardalan et al. emphasize the importance of a personalized dietary approach based on pouch phenotype. While patients with a normal or irritable pouch may benefit primarily from strategies targeting gastrointestinal function, those with risk factors for pouchitis or established recurrent/chronic pouchitis may require dietary interventions that specifically address the microbial and immunological mechanisms implicated in disease pathogenesis [23].

### 6.5. Faecal Microbiota Transplantation

The rationale for fecal microbiota transplantation (FMT) in pouchitis rests on the premise that transferring a microbial community from a healthy donor can restore eubiosis, replenish depleted protective taxa, and re-establish colonization resistance against pro-inflammatory pathobionts [97]. FMT has demonstrated efficacy in recurrent *Clostridioides difficile* infection, providing proof of principle that microbial community replacement can resolve dysbiosis-driven disease [98].

Three small RCTs have evaluated FMT for pouchitis, both with negative results [99,100]. Herfarth et al. (2019) assessed FMT for maintenance of remission in antibiotic-dependent pouchitis over a 4-month period [99]. FMT was delivered directly to the pouch via endoscopy, followed by oral capsules for 2 weeks [99]. The trial was stopped prematurely after only 6 patients were enrolled due to lower-than-expected clinical remission rates and low microbial engraftment [99]. No participant in either the FMT or placebo group was able to maintain remission over the course of the study, though FMT was well tolerated with no FMT-related adverse events [99]. Karjalainen et al. (2021) assessed maintenance of remission over a 12-month period, comparing donor FMT with autologous FMT [100]. FMT was delivered directly to the pouch via endoscopy, followed by a single FMT treatment given via transanal catheter. There was no statistically significant difference in the proportion of participants maintaining clinical remission: 31% (4/13) of FMT participants versus 38% (5/13) of placebo participants (RR 0.80; 95% CI 0.28–2.32; GRADE low certainty evidence) [100]. Notably, individuals on continuous antibiotics before FMT in the donor FMT arm had a significantly higher rate of relapse than those who received autologous FMT, suggesting that prior antibiotic exposure may paradoxically impair FMT engraftment [99,100]. More recently, Kousgaard et al. (2024) conducted the multicentre, randomized, double-blind, placebo-controlled MicroPouch trial in 30 patients with chronic pouchitis [101]. Participants received non-pooled multidonor FMT or placebo by enema daily for 2 weeks and every second day for an additional 2 weeks [101]. After the 4-week intervention period, FMT was not superior to placebo for inducing clinical remission, with no significant difference in remission rates between groups (RR 1.0) [101]. Although metagenomic analyses demonstrated significantly greater donor microbiota engraftment in the FMT group, these microbiome changes did not translate into clinical benefit [101]. Adverse events were more frequent among FMT recipients, but no new safety concerns were identified [101], with temporary worsening of symptoms and decreased quality of life (QoL) [102]. Collectively, these trials suggest that, despite evidence of microbial engraftment, FMT has not demonstrated efficacy for either induction or maintenance of remission in chronic pouchitis. Yet, these studies were limited by the lack of a validated instrument for measuring pouchitis disease activity, small sample sizes, and heterogeneous patient populations [4].

Despite the negative RCT results, observational data have suggested a more favorable signal, though with significant heterogeneity. In a systematic review and meta-analysis including 103 patients, Zaman et al. reported pooled clinical response and remission rates of 42.6% and 29.8%, respectively [103]. FMT was generally well tolerated, with adverse events being mild and self-limiting, and no major safety concerns identified during either short- or long-term follow-up [103]. Levels of donor microbiota engraftment varied considerably between studies [103]. Patients with pouchitis typically exhibited reduced microbial richness and diversity, and some investigations documented shifts toward a microbiome composition more closely resembling that of healthy pouches following FMT [103]. Similar findings were reported by Chun et al., who analyzed seven studies comprising 94 patients [104]. The pooled estimates showed an overall remission rate of 15%, a clinical response rate of 33%, a clinical remission rate of 14%, and a clinical relapse rate of 36% [104]. Although mild adverse events occurred in a proportion of participants, no severe treatment-related complications or deaths were observed [104]. Consistent with these findings, a systematic review of single-arm observational studies referenced in the AGA guideline reported a pooled remission rate of 20.1% among patients with chronic pouchitis receiving conventional FMT, a figure comparable to remission rates observed in placebo groups of randomized trials [4].

Additional insight was provided by Kousgaard et al. in a prospective open-label pilot study involving nine patients with chronic pouchitis who received enema-administered FMT from five donors over a 14-day period [105]. Clinical remission was achieved by four participants at 30 days, with three maintaining remission at six months [105]. While symptom severity improved significantly during follow-up, corresponding improvements in overall PDAI scores were not observed [105]. Importantly, six patients demonstrated substantial microbiome restructuring after FMT, including increased microbial diversity and successful engraftment of donor-derived taxa [105]. This dissociation between microbial engraftment and clinical response suggests that successful microbial colonization alone may be insufficient for clinical benefit.

However, the ileal pouch presents unique biological challenges that distinguish it from the colon, the anatomical site where FMT has been most successful. The pouch environment is characterized by lower pH, higher oxygen tension, altered bile acid composition, and fecal stasis, all of which may impede engraftment of donor-derived colonic microbiota [4]. Given the unique characteristics of the ileal pouch, the AGA has suggested that donor microbiota engraftment may differ from that observed in the colon [4]. This raises uncertainty regarding the appropriateness of using predominantly colonic microbial communities for FMT in pouchitis [4]. This fundamental biological mismatch between donor microbiota (colonic) and recipient environment (ileal pouch) may explain the disappointing clinical results observed to date [106].

To overcome this potential ecological mismatch between colonic donor microbiota and the ileal pouch environment, Kousgaard et al. (2025) conducted a proof-of-concept study evaluating FMT derived from a donor with a normally functioning IPAA in patients with chronic pouchitis [107]. Three patients received FMT by enema daily for 2 weeks, then every other day for 2 more weeks. At 30-day follow-up, all 3 participants achieved clinical remission with reduced endoscopic inflammation, with the median total PDAI score decreasing from 8 to 6 [107]. Two participants reported improved quality of life. Adverse events were mild and self-limited. Metagenomic analysis revealed distinct microbiome clusters between the IPAA donor and recipients, both differing from those of healthy donors [107]. While extremely preliminary (*n* = 3), this approach addresses the fundamental biological concern that colonic microbiota may be poorly suited for engraftment in an ileal pouch and suggests that pouch-adapted donor microbiota may be more appropriate.

Due to the limited number of studies, identifying predictors of FMT response in pouchitis remains an unresolved challenge, though several factors have emerged from the available data. Donor selection is important as the concept of “super-donors”, individuals whose microbiota is particularly effective at engrafting and inducing clinical benefit, has been explored in FMT for UC but has not been systematically studied in pouchitis [103]. The AGA has noted that engraftment may depend on donor source, including individuals with healthy colons, microbiota from the small bowel, or individuals with ileal pouches without pouchitis [4]. The proof-of-concept study by Kousgaard et al. using a healthy pouch donor suggests that donor–recipient ecological compatibility may be a critical determinant of success [107]. Additionally, recipient-related factors may play an important role. For example, prior antibiotic exposure appears to influence FMT outcomes. In the Karjalainen et al. RCT, individuals on continuous antibiotics before FMT in the donor FMT arm had a significantly higher rate of relapse than those who received autologous FMT [100]. This finding is consistent with the observation by Dubinsky et al. that antibiotic treatment establishes an antibiotic-resistant microbiome that may resist displacement by donor organisms [62]. FMT outcomes may differ according to pouchitis phenotype and treatment history, with disease chronicity and prior responsiveness to antibiotics emerging as potential determinants of therapeutic response [4]. Furthermore, successful FMT engraftment in recipients has varied across studies [103]. Kousgaard et al. demonstrated that FMT increased microbial diversity in 6 of 9 patients, with high engraftment of specific donor microbiota, but clinical remission was achieved in only 4 of 9 [108]. This suggests that engraftment is necessary but not sufficient for clinical benefit and that additional host factors, including immune status, barrier integrity, and genetic susceptibility, modulate the clinical response to microbial community replacement. Finally, the optimal route, preparation, quantity, and frequency of FMT for pouchitis remain undefined [103]. Studies have used endoscopic delivery directly to the pouch, transanal catheters, enemas, and oral capsules, with no standardized protocol [4,103]. The AGA has emphasized that FMT delivery route and treatment intervals are likely important determinants of engraftment and efficacy [4]. The unique anatomy of the ileal pouch, often with a J-configuration, reduced surface area, and altered motility, may require pouch-specific delivery strategies distinct from those used for colonic FMT.

Together, current evidence does not support the routine use of FMT for pouchitis [103]. Randomized controlled trials have failed to show a significant benefit over placebo, and current guidelines recommend its use only within clinical trials. Key limitations include poor efficacy, challenges with microbiota engraftment in the ileal pouch environment, the absence of validated disease activity endpoints, substantial heterogeneity in FMT protocols, and limited understanding of recipient-related factors that influence response. Although FMT appears generally safe, long-term safety data remain limited, and regulatory considerations continue to evolve. Future well-designed, adequately powered trials, together with mechanistic studies of the pouch microbiome and engraftment strategies, are needed before FMT can be considered a viable treatment option for pouchitis.

## 7. Challenges and Research Gaps

### 7.1. The Antibiotic Paradox

One of the most intriguing observations in pouchitis research is the apparent disconnect between clinical response and microbial restoration following antibiotic therapy. Although antibiotics are highly effective in inducing remission, increasing evidence suggests that they do not restore a healthy microbial ecosystem and may instead further alter microbial community structure [67]. This phenomenon has been termed the antibiotic paradox and challenges the traditional assumption that therapeutic efficacy results primarily from eradication of pathogenic bacteria [12,62].

Reshef et al. demonstrated that antibiotic treatment was associated with reduced abundance of multiple bacterial genera and increased relative abundances of Enterococcus and Enterobacteriaceae, suggesting that clinical improvement may occur despite persistence or even worsening of microbial dysbiosis [25]. Further insight was provided by Dubinsky et al., who performed shotgun metagenomic analyses of 234 fecal samples from 49 pouch patients [62]. Although 79% of patients responded clinically to antibiotic treatment, antibiotic-responsive microbiomes were dominated by facultative anaerobic genera, including Escherichia, Enterococcus, and Streptococcus, many of which carried ciprofloxacin-resistance mutations [62]. Importantly, these antibiotic-resistant strains exhibited reduced virulence potential and failed to induce inflammatory cytokine secretion by epithelial cells, suggesting that antibiotics may promote the establishment of a microbial community with lower inflammatory capacity rather than restore eubiosis [62]. Despite these short-term benefits, relapse after treatment is common. Among patients completing a four-week antibiotic course, 89% relapsed within three months of antibiotic cessation [62]. This recurrence was accompanied by abrupt shifts in microbiome composition and blooms of oral and disease-associated bacteria, supporting the notion that antibiotic therapy suppresses rather than permanently corrects the underlying microbial disturbance [62].

Antibiotic exposure also has important consequences for antimicrobial resistance. Dubinsky et al. demonstrated enrichment of strains carrying multidrug-resistance loci, including genes encoding extended-spectrum β-lactamases [62]. Consistent with these findings, ESBL-producing bacteria have been identified in approximately one-third of patients with recurrent or refractory pouchitis and prepouch ileitis [40]. While long-term clinical studies have not demonstrated increased rates of resistant infections among pouch patients receiving chronic antibiotic therapy, the emergence and persistence of resistant microbial communities remain a concern [72].

Taken together, these findings suggest that antibiotic efficacy in pouchitis is more complex than simple pathogen eradication. Rather than restoring a healthy microbial ecosystem, antibiotics may transiently suppress highly inflammatory microbial communities while selecting for alternative communities with lower virulence potential. Understanding how to achieve durable remission without promoting antimicrobial resistance, therefore, remains one of the central challenges in the management of pouchitis.

### 7.2. Methodological Limitations

The interpretation of microbiota studies in pouchitis is complicated by significant methodological heterogeneity across the literature [15,21]. Techniques used to assess microbial diversity range from conventional culture-based methods to culture-independent sequencing approaches [15,21]. Culture-based analyses may skew results toward aerobically cultivable organisms, potentially underrepresenting anaerobic taxa that are important constituents of the pouch microbiome [21]. Although 16S rRNA sequencing largely circumvents cultivation bias, it does not differentiate between living and dead bacterial cells and therefore may not accurately reflect microbial viability [21]. Shotgun metagenomics provides the most comprehensive functional and taxonomic resolution but has been applied in only a limited number of studies [20]. Additional sources of heterogeneity include differences in sampling strategies (fecal vs. mucosal biopsies), patient populations (acute vs. chronic pouchitis, antibiotic-naive vs. antibiotic-exposed), disease activity assessment methods, and bioinformatic pipelines [15,21]. Interpretation is further complicated by the dynamic nature of the pouch ecosystem as most studies are cross-sectional. Thus, they cannot evaluate whether a given taxonomic shift is a cause of inflammation, a consequence of it, or a compensatory response. In particular, the relative enrichment of certain commensal or putatively anti-inflammatory taxa may not be pathogenic but could instead reflect a reactive attempt to suppress localized inflammation and restrain pathobionts, for example, through bacteriocin production, meaning that a single organism may carry different significance depending on the ecological and inflammatory context. The lack of standardized microbiota assessment methods across studies limits comparability and reproducibility, and a formal meta-analysis of microbiota data has not been attempted due to this heterogeneity [15]. Future studies should adopt standardized protocols for sample collection, processing, sequencing, and bioinformatic analysis and ideally longitudinal, functional designs that can distinguish cause, consequence, and compensation, to enable meaningful cross-study comparisons.

### 7.3. More than a Microbiota-Driven Disorder

Although accumulating evidence implicates the gut microbiota in pouchitis pathogenesis, an alternative interpretation is that pouchitis is not solely a microbiota-driven disorder but rather the result of complex interactions among microbial, immunological, genetic, epithelial, and environmental factors [13]. Braga-Neto et al. argued that pouchitis should be viewed as “more than just the microbiota”, emphasizing that many observed microbial alterations may represent consequences rather than causes of inflammation and that host-specific factors likely play a central role in determining disease susceptibility and progression [59]. This perspective is supported by several observations, including the predominance of cross-sectional studies, the inconsistency of microbial signatures across cohorts, and the variable efficacy of microbiota-targeted interventions.

These competing interpretations are not necessarily mutually exclusive. Rather than acting as a sole driver of disease, microbial dysbiosis may interact with defects in epithelial barrier function, altered immune responses, genetic susceptibility, and environmental exposures to influence disease onset and course. In this framework, the microbiota functions as one component of a multifactorial pathogenic network, acting as both a modulator and amplifier of inflammatory processes.

Distinguishing between these models remains a major research priority. Evidence supporting a predominantly causal role would require adequately powered prospective studies demonstrating that specific microbial alterations consistently precede disease development and predict subsequent pouchitis. Conversely, evidence that microbial changes occur only after inflammation is established would support the view that dysbiosis is primarily a downstream consequence of disease. Interventional studies showing that targeted restoration of microbial composition or function leads to sustained endoscopic remission would provide the strongest evidence for causality. Until such data become available, the microbiota should be regarded as a likely contributor to pouchitis pathogenesis within a broader host–microbe disease framework rather than as the sole determinant of disease.

### 7.4. Strengths and Limitations of This Review

A major strength of this review is its integration of mechanistic, microbiome, and clinical evidence to provide a comprehensive overview of the role of the gut microbiota in pouchitis. The review also incorporates recent advances in microbiome science, including emerging therapeutic and precision medicine approaches. However, several limitations should be acknowledged. As a narrative review, it did not include a formal risk-of-bias assessment or quantitative synthesis. In addition, the conclusions are constrained by the heterogeneity of the available literature and the predominance of small studies in several areas of pouchitis research.

## 8. Future Perspectives

The increasing recognition of pouchitis as a microbiota-associated disorder has stimulated the development of next-generation therapeutic and diagnostic approaches that extend beyond conventional antibiotics and probiotics. Advances in microbiome science, systems biology, and bioengineering are creating new opportunities to both understand and manipulate the pouch ecosystem with greater precision (Box 1).

Rationally designed bacterial consortia offer standardized composition, improved safety, and regulatory tractability compared to FMT [109,110]. Several are in clinical development for IBD: VE202 (spore-forming Clostridia, phase 2 for UC); MH002 (six-member consortium, phase 2a showing 42% calprotectin reduction); and SER-287 (Firmicutes spores, 40% vs. 0% remission in phase 1, though the subsequent SER-301 trial was halted for futility) [110,111]. Preclinical consortia GUT-103 and GUT-108 cured experimental colitis through metabolite-dependent mucosal healing [110]. While none have been evaluated in pouchitis, a pouch-specific consortium enriched in *F. prausnitzii*, *Roseburia* spp., and *Blautia* spp. could represent a rational therapeutic strategy.

Beyond the administration of defined microbial communities, genetically engineered bacteria by synthetic biology approaches enable microenvironment-responsive therapeutic targeting, incorporating sense-and-respond circuits that detect inflammatory signals and deliver payloads such as anti-TNFα nanobodies, IL-10, or trefoil factors at sites of inflammation [112,113]. Similarly, bacteriophage-based therapies could provide highly selective suppression of pathobionts such as *Klebsiella pneumoniae*, *Clostridium perfringens*, *Ruminococcus gnavus*, and *Fusobacterium* spp. while avoiding the broad ecological disruption associated with antibiotics [114,115]. Additional approaches under investigation include microbial metabolites, bacterial membrane vesicles, and bacterial function-editing substrates designed to reproduce beneficial microbiome functions without requiring durable microbial engraftment [109]. However, several challenges remain including long-term safety, genetic stability, containment, delivery to the ileal pouch, bacterial resistance, strain-level target identification and regulatory compliance [112,113].

Beyond therapeutic development, improved experimental models are likely to accelerate mechanistic discoveries in pouchitis. Intestinal organoids derived from pouch biopsies, organoid-on-chip systems, and CRISPR/Cas9-based genetic manipulation provide opportunities to investigate epithelial barrier dysfunction, host–microbiota interactions, and treatment responses in patient-specific settings [116,117]. Furthermore, three-dimensional bioengineered tissue models may facilitate the study of interactions among epithelial, immune, stromal, and microbial compartments within a controlled environment [118].

Complementing these experimental approaches, the integration of multi-omics technologies, including metagenomics, metatranscriptomics, metabolomics, proteomics, and epigenomics, is poised to transform pouchitis research [119,120]. These approaches provide insight not only into microbial composition but also into microbial function, host responses, and disease-specific molecular pathways. Future studies incorporating strain-level microbial profiling, antimicrobial resistance characterization, and functional pathway analyses may identify novel biomarkers and therapeutic targets [20,62]. Ultimately, these advances support the development of precision medicine approaches for pouchitis. Integration of microbial signatures, metabolic profiles, host genetic susceptibility, and clinical characteristics may allow identification of patients at risk for disease development, prediction of treatment responses, and selection of individualized therapeutic strategies [22,59,62,109,110,121]. Artificial intelligence and machine learning approaches will likely play an increasingly important role in translating complex multi-omic datasets into clinically actionable decision-support tools [109,121].

Box 1 **Overview of key take-home messages and research priorities.**

**Key take-home messages**

1. Pouchitis is associated with microbial dysbiosis characterized by reduced microbial diversity, depletion of beneficial anaerobic commensals, and altered microbial metabolic pathways.
  Certainty: Moderate.
2. Current evidence supports a contributory, and probably causal, role of the gut microbiota in pouchitis pathogenesis, although definitive causality has not yet been established.
  Certainty: Low to moderate.
3. Antibiotics remain the cornerstone of treatment and are effective in many patients, but they do not restore microbial eubiosis and are associated with frequent relapse.
  Certainty: Moderate to high.
4. Among microbiota-targeted interventions, the eight-strain De Simone formulation currently has the strongest evidence for pouchitis prevention, whereas fecal microbiota transplantation remains investigational and is not supported for routine clinical use outside research settings.
  Certainty: Moderate.
5. Diet and nutrition represent promising but underexplored modulators of the pouch microbiota, with potential implications for disease prevention and treatment that require further study.
  Certainty: Low.

**Research priorities**

1. Prospective longitudinal studies to identify microbial, metabolic, and dietary predictors of pouchitis development and recurrence.
2. Randomized controlled trials of microbiota-targeted interventions, including dietary strategies, live biotherapeutics, and microbial replacement therapies, using standardized endoscopic and clinical outcomes.
3. Multi-omics studies integrating microbiome, metabolome, immune, and host genetic data to support patient stratification and the development of personalized microbiome-based therapies.

## 9. Conclusions

The available evidence indicates that pouchitis is characterized by reduced microbial diversity, depletion of beneficial anaerobic commensals, enrichment of potentially pro-inflammatory taxa, and alterations in key metabolic pathways. These microbial changes are consistently associated with pouch inflammation and represent the most reproducible microbiological feature of the disease. Mechanistically, the microbiota may contribute to pouch inflammation through disrupted barrier function, altered microbial metabolite production, and dysregulated host immune responses, although the relative contribution of these pathways remains incompletely defined.

Current evidence supports a contributory, and probably causal, role of the gut microbiota in pouchitis pathogenesis. This conclusion is supported by characteristic microbial signatures associated with disease activity, associations between pre-operative microbial profiles and subsequent pouchitis risk, and the clinical responsiveness of many patients to microbiota-targeted interventions. However, much of the available evidence remains associative, and definitive causal relationships have not yet been fully established. Moreover, although the microbiota represents an attractive therapeutic target, evidence supporting microbiota-directed interventions remains limited. While antibiotics are effective in many patients, other approaches such as fecal microbiota transplantation have yielded inconsistent results, including negative randomized controlled trials, highlighting the need for more robust clinical evidence. To better determine the extent to which microbial alterations drive disease initiation and progression, future research should include adequately powered prospective cohort studies with pre-specified microbial predictors, alongside interventional studies evaluating microbial replacement or modulation strategies using objective endoscopic and clinical endpoints.

From a therapeutic perspective, antibiotics remain the backbone of treatment, although they do not restore microbial eubiosis and are limited by frequent relapse and concerns regarding antimicrobial resistance, highlighting the need for more durable microbiota-directed therapies. Among microbiota-targeted approaches, the strongest evidence currently supports the eight-strain De Simone formulation for prevention, whereas evidence for fecal microbiota transplantation remains inconsistent and does not support its routine use outside clinical trials. Diet and nutrition represent promising, non-pharmacological approaches for modulating the pouch microbiota, although high-quality prospective and interventional studies are needed before dietary strategies can be incorporated into routine pouchitis management.

Advances in multi-omics technologies, precision microbiome medicine, next-generation live biotherapeutics, and microbiota-targeted interventions have the potential to fundamentally transform the prevention and management of pouchitis. Continued integration of microbial, metabolic, immunological, and genetic data will be essential for developing personalized therapeutic strategies capable of achieving sustained remission and improving long-term patient outcomes.

## Figures and Tables

**Table 1:** Overview of changes in microbiota in pouchitis.

Taxon or Microbial Group	Change	Relevance
*Clostridium perfringens*	↑	Mucin-degrading organism repeatedly associated with pouchitis and predictive of disease development [22,23].
Enterobacteriaceae (including *Escherichia coli*)	↑	Bacteria with invasive properties that can generate potentially harmful metabolites [21,23].
*Fusobacterium* spp.	↑	Associated with hydrogen sulphide production; correlated with disease activity and CRP levels [23,25].
Sulphate-reducing bacteria (*Desulfovibrio*, *Bilophila*)	↑	More abundant in UC pouches and during active pouchitis; contribute to hydrogen sulphide production [18,23].
*Ruminococcus gnavus*	↑	Mucin-degrading organism enriched in pouchitis; strains encoding glycoside hydrolases are overrepresented [20,23].
*Ruminococcus obeum*	↑	Associated with pouchitis in microbial profiling studies [18].
*Bacteroides vulgatus*	↑	Enriched in patients who subsequently develop pouchitis [22].
Total aerobic bacteria	↑	Reflects expansion of aerobic and facultative anaerobic populations during inflammation [18].
*Faecalibacterium* spp. (*F. prausnitzii*)	↓	Major butyrate producer with anti-inflammatory properties; inversely correlated with CRP levels [23,25].
Lachnospiraceae (*Blautia*, *Roseburia*)	↓	Important butyrate producers and key bacteria involved in secondary bile acid formation [22,23].
Ruminococcaceae	↓	Beneficial anaerobic commensals involved in SCFA production and intestinal homeostasis [23].
*Bifidobacterium* spp.	↓	Suppress potentially pathogenic bacteria and promote butyrate production through cross-feeding [23].
*Lactobacillus* spp.	↓	Beneficial commensals associated with intestinal homeostasis [18].
*Streptococcus* spp.	↓	Reduced abundance reported in pouchitis-associated dysbiosis [18].
Enterococcaceae	↓	Depleted in patients with pouchitis [20].
Alcaligenaceae	↓	Reduced abundance in pouchitis [21].
*Eubacterium rectale*	↓	Important butyrate-producing species depleted during pouchitis [21].
*Parabacteroides* spp.	↓	Reduced abundance during pouchitis [18].
Total anaerobic bacteria	↓	Reflects loss of beneficial obligate anaerobes and reduced anaerobic-to-aerobic ratio [18].

## Data Availability

No new data were created or analyzed in this study. Data sharing is not applicable to this article.
